# Epidemiological characteristics of holoprosencephaly in China, 2007-2014: A retrospective study based on the national birth defects surveillance system

**DOI:** 10.1371/journal.pone.0217835

**Published:** 2019-06-06

**Authors:** Ling Yi, Zhongqiang Liu, Changfei Deng, Xiaohong Li, Ke Wang, Kui Deng, Yi Mu, Jun Zhu, Qi Li, Yanping Wang, Li Dai

**Affiliations:** 1 National Center for Birth Defects Monitoring, West China Second University Hospital, Sichuan University, Chengdu, Sichuan, China; 2 Key Laboratory of Birth Defects and Related Diseases of Women and Children (Sichuan University), Ministry of Education, Chengdu, Sichuan, China; 3 Department of Pediatric Intensive Care Unit, West China Second University Hospital, Sichuan University, Chengdu, Sichuan, China; 4 Medical Big Data Center, Sichuan University, Chengdu, Sichuan, China; Ohio State University, UNITED STATES

## Abstract

**Objective:**

To describe the epidemiology of holoprosencephaly (HPE) in China with special reference to prevalence and associated anomalies.

**Methods:**

Data were abstracted from the Chinese Birth Defects Monitoring Network for the period 2007–2014. Birth prevalence of HPE were assessed by birth year, fetal/infant sex, maternal age, and maternal residential area. Poisson regressions were used to calculate the crude and adjusted prevalence ratios (PR) and their 95% confidence intervals, and linear chi-square test was used to explore time trend for the prevalence of HPE.

**Results:**

A total of 1222 HPE cases were identified in 13,284,142 births, yielding an overall prevalence of 0.92 per 10,000 births. The annual prevalence of HPE presented an upward trend (*P*<0.001), from 0.54 per 10,000 births in 2007 to 1.21 per 10,000 births in 2014. Higher prevalence was found in older maternal-age groups (30–34 years, adjusted PR: 1.19, 95% CI: 1.02–1.40; ≥35 years, adjusted PR: 1.53, 95% CI: 1.26–1.86) in comparison with the maternal-age group of 25 to 29 years. Higher prevalence was also found in infants born to mothers resided in urban areas (adjusted PR: 1.23, 95% CI: 1.08–1.39) and female infants (adjusted PR: 1.30, 95% CI: 1.15–1.47).

**Conclusions:**

HPE is an important perinatal health issue because of its poor prognosis. This is the first study depicting a picture of epidemiological characteristics of HPE in China, which can provide useful references for future studies.

## Introduction

Holoprosencephaly (HPE) is a complex developmental disorder of the human forebrain resulting from incomplete separation of the prosencephalon into right and left hemispheres [1–4]. HPE can be categorized into alobar, semilobar, lobar and syntelencephaly types according to degree of hemispheric separation. Typical HPE is usually accompanied by one or more facial anomalies such as cyclopia, proboscis, microcephaly, hypotelorism, midface hypoplasia, cleft lip and/or palate. A number of environmental factors have been linked with HPE, including cytomegalovirus infection, prenatal exposure to drugs, maternal diabetes and alcoholism [5]. Gene mutations [6–9] and chromosome anomalies (particularly trisomy 13,18 and 21) have been identified in HPE patients. Notably, about 65% of HPE cases were sporadic, and their causes remain to be elucidated [1]. The prognosis varies in each type of HPE, principally depending on the severity and associated complications [10, 11]. Children with alobar HPE or with severe facial malformations (i.e. cyclopia and proboscis) rarely survive the neonatal period, while those with less severe facial anomalies can survive for months. Few HPE cases can survive into adulthood.

HPE occurs in about 1 out of 250 conceptuses [12]. However, the prevalence at birth is much lower because most affected embryos are eliminated by spontaneous abortion during gestation. The reported birth prevalence varied by countries and regions, ranging from 0.48 to 1.70 per 10,000 births [13–19]. The information on prevalence, associated anomalies and outcome of HPE is of great importance both from epidemiological and clinical perspective. The existing studies on HPE prevalence were mostly conducted in the United States and some European countries [13–19].

In China, nervous system malformations have received considerable attention due to their poor prognosis and high prevalence [20, 21]. Starting in 2009, the Ministry of Health of China initiated a nationwide program of folic acid supplementation to prevent neural tube defects, and good intervention effect has been achieved [22, 23]. Other nervous system malformations, such as HPE, have become a rising concern. This study aims to provide an epidemiologic profile of HPE in Chinese population, using data from the Chinese Birth Defects Monitoring Network (CBDMN) between 2007 and 2014.

## Methods

### Study subjects

The CBDMN is a nationwide birth defects surveillance program that was set up in 1986 and now is administered by National Health Commission. It is based on reports from approximately 780 member hospitals throughout China. At present more than two million births are covered per year, representing over 10% of Chinese live births. For each member hospital the collection and reporting of data is mandatory. At the beginning of CBDMN only birth defects diagnosed within perinatal period (from 28 weeks of gestation to 7 days after birth) were reported. Since 2003, the year when the Administrative Method on Antenatal Diagnostic Techniques regulation was issued [24], any case regardless of gestational age, either live or terminated but confirmed within 7 days after birth has been eligible for inclusion. According to the regulation, all pregnant women are encouraged to have the first and second trimester screening and possible diagnosis for congenital anomalies. Diagnosis of congenital malformation is usually made by obstetrics, pediatrics or ultrasound experts at member hospitals. The details of case ascertainment in the monitoring system have been described elsewhere [25–27].

All anomalies in CBDMN database are coded by the International Classification of Disease version 10 (ICD10). Specifically, diagnostic measures commonly used to identify HPE include ultrasound sonography, computer tomography and magnetic resonance imaging. CBDMN adopts the criteria of HPE cases proposed by the International Clearinghouse for Birth Defects Surveillance and Research (ICBDSR) [28], including cyclopia, ethmocephaly, cebocephaly, and premaxillary agenesis diagnosed prenatally or within the first week of life. Infants and fetuses with the ICD10 code for HPE (Q04.2) were searched in the CBDMN database from January 2007 to December 2014.

### Statistical analysis

The birth prevalence was calculated as the number of HPE cases per 10,000 live and still births. Linear chi-square test was conducted to assess the time trends of prevalence over the study period [29]. Poisson regressions were performed to calculate the crude and adjusted prevalence ratios (PR) and their 95% confidence interval (95%CI). When computing the adjusted PRs by one of these factors (birth year, fetal/infant sex, maternal age, and maternal residential area), we controlled the effects of others. Maternal age was divided into five age groups: <20 years, 20–24 years, 25–29 years, 30–34 years and ≥35 years. Maternal residential area was categorized as urban (cities, urbanized areas or neighborhood communities) or rural (villages or countryside) area, according to the last residence of the mother for at least 1 year. The rules of urban-rural classification in the CBDMN conform to the regulation released by National Bureau of Statistics of China [30]. All statistical analyses were performed using the SPSS 21.0 program. The statistical significance level for α was set at 0.05.

## Results

**Fig 1:**
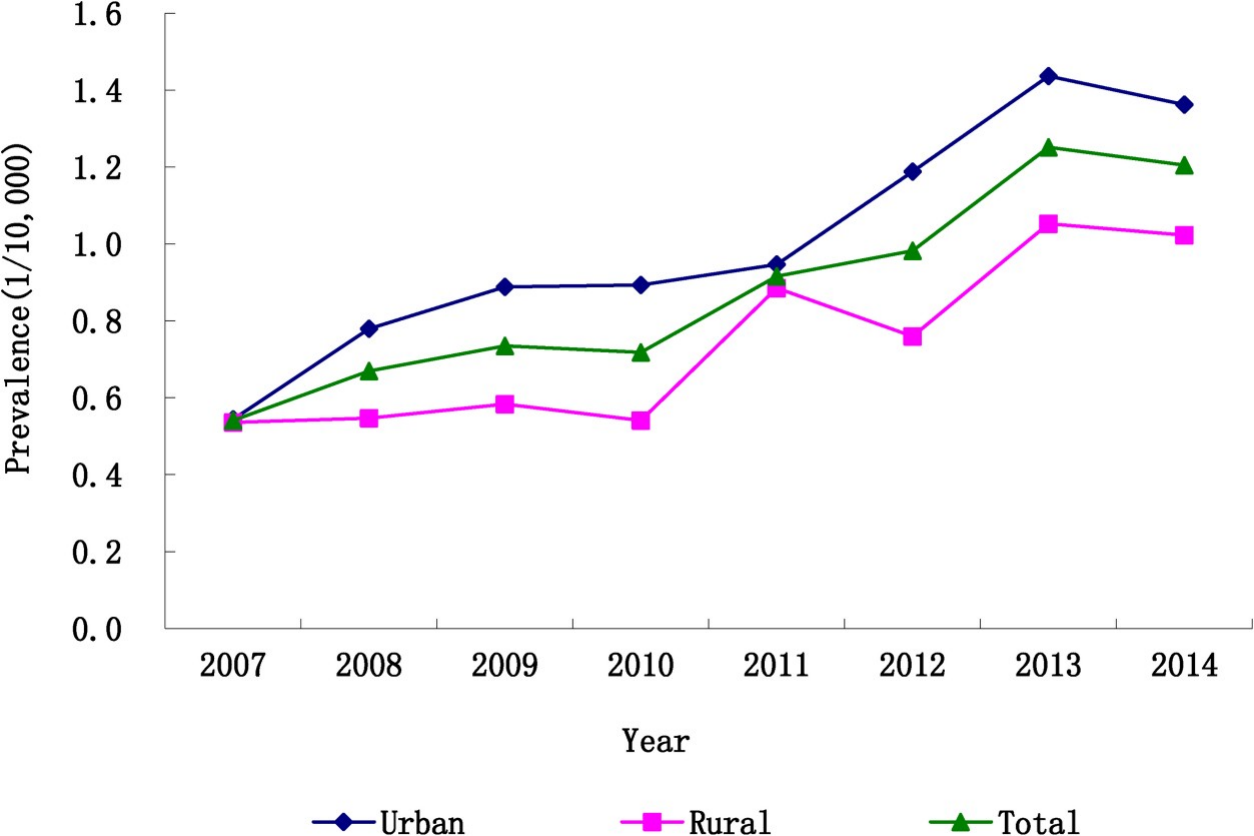
Time trends in prevalence of HPE in China, 2007–2014.

During 2007 to 2014, a total of 1222 cases with HPE were identified in CBDMN, comprising 1186 singleton pregnancies and 36 twin pregnancies (34 twin individuals, and one pair of twins). There were 13,284,142 registered births during the study period, giving a total prevalence of 0.92 (95% CI: 0.87–0.97) per 10,000 births. The prevalence of HPE presented an upward time trend during the period of 2007–2014 (Fig 1), from 0.54 to 1.21 per 10,000 births for the total HPE (*P*<0.001), from 0.67 to 1.35 per 10,000 births for HPE in the urban areas (*P*<0.001) and from 0.41 to 1.06 per 10,000 births for HPE in the rural areas (*P*<0.001).

The prevalence of HPE for selected demographic factors were listed in Table 1. Higher prevalence was observed in older maternal-age groups (30–34 years, adjusted PR: 1.19, 95% CI: 1.02–1.40; ≥35 years, adjusted PR: 1.53, 95% CI: 1.26–1.86), infants born to mothers resided in urban areas (adjusted PR: 1.23, 95% CI: 1.08–1.39) and female infants (adjusted PR: 1.30, 95% CI: 1.15–1.47).

**Table 1:** The crude and adjusted prevalence ratios of HPE by birth year, fetal/infant sex, maternal age, and maternal residential area in China, 2007–2014.

	HPE cases	Total births	Prevalence per 10,000 births (95%CI)	Crude PR (95%CI)	Adjusted PR (95%CI)
Birth year					
2007	68	1258298	0.54 (0.41–0.67)	1.00 (reference)	1.00 (reference)
2008	88	1314076	0.67 (0.53–0.81)	1.19 (0.86–1.64)	1.19 (0.86–1.65)
2009	103	1401331	0.74 (0.59–0.88)	1.27 (0.92–1.73)	1.27 (0.93–1.74)
2010	110	1531143	0.72 (0.58–0.85)	1.16 (0.84–1.59)	1.16 (0.85–1.59)
2011	154	1681096	0.92 (0.77–1.06)	1.56 (1.17–2.10)	1.57 (1.17–2.10)
2012	197	2005526	0.98 (0.85–1.12)	1.64 (1.24–2.18)	1.65 (1.24–2.19)
2013	237	1893854	1.25 (1.09–1.41)	1.99 (1.51–2.63)	1.99 (1.50–2.63)
2014	265	2198818	1.21 (1.06–1.35)	1.85 (1.40–2.43)	1.84 (1.40–2.43)
Maternal age (years)					
<20	27	287013	0.94 (0.59–1.30)	1.22 (0.81–1.83)	1.27 (0.85–1.91)
20–24	274	3339333	0.82 (0.72–0.92)	1.06 (0.91–1.25)	1.14 (0.97–1.34)
25–29	469	5581574	0.84 (0.76–0.92)	1.00 (reference)	1.00 (reference)
30–34	288	2856847	1.01 (0.89–1.12)	1.20 (1.03–1.41)	1.19 (1.02–1.40)
≥35	164	1219375	1.34 (1.14–1.55)	1.51 (1.24–1.84)	1.53 (1.26–1.86)
Maternal residential area					
Urban	729	6914454	1.05 (0.98–1.13)	1.22 (1.08–1.37)	1.23 (1.08–1.39)
Rural	493	6369688	0.77 (0.71–0.84)	1.00 (reference)	1.00 (reference)
Fetal/infant sex					
Male	492	7061415	0.70 (0.64–0.76)	1.00 (reference)	1.00 (reference)
Female	564	6219683	0.91 (0.83–0.98)	1.30 (1.15–1.47)	1.30 (1.15–1.47)

Among 1222 HPE cases, 236 (19.3%) cases were isolated HPE, and 986 (80.7%) HPE cases were accompanied with additional anomalies (chromosomal or other structural malformations). 1154 (94.4%) HPE cases were terminations of pregnancy, 55 (4.5%) HPE cases were live births, and 13 (1.1%) HPE cases were stillbirths. 29 HPE cases died within the first 7 days after birth. The early neonatal mortality rates (ENMR) were 33.3% and 56.5% for isolated HPE and HPE accompanied with additional anomalies, respectively. Table 2 shows the additional malformations present in HPE cases. The most common additional anomalies were circulatory system malformation, presenting in 20.6% of cases. Musculoskeletal and respiratory system anomalies occurred in 18.8% and 17.5% of cases, respectively. 17.0% cases had cleft lip and/or palate. Only 1.6% of cases were accompanied by chromosomal abnormalities.

**Table 2:** Abnormalities associated with HPE.

System/Abnormalities	ICD10 code	HPE cases	%
Nervous system^*^	**Q00-Q07**	**92**	**7.5**
Anencephaly	Q00	25	2.0
Hydrocephalus	Q03	78	6.4
Spina bifida	Q05	33	2.7
**Eye, ear, face and neck**	**Q10-Q18**	**112**	**9.2**
Anophthalmos, microphthalmos	Q11.0- Q11.2	10	0.8
Cyclopia	Q11.4	33	2.7
Malformations of ear	Q17	25	2.0
Malformations of face and neck	Q18	26	2.1
**Circulatory system**	**Q20-Q28**	**252**	**20.6**
Malformations of cardiac chambers and connections	Q20	42	3.4
Malformations of cardiac septa	Q21	152	12.4
Malformations of peripheral circulatory system	Q27	31	2.5
**Respiratory system**	**Q30-Q34**	**214**	**17.5**
Malformations of nose ^**#**^	Q30	213	17.4
**Cleft lip and cleft palate**	**Q35-Q37**	**208**	**17.0**
Cleft palate	Q35	4	0.3
Cleft lip	Q36	65	5.3
Cleft palate with cleft lip	Q37	139	11.4
**Digestive system**	**Q38-Q45**	**0**	**0.0**
**Genital organs**	**Q50-Q56**	**17**	**1.4**
**Urinary system**	**Q60-Q64**	**55**	**4.5**
Cystic kidney disease	Q61	14	1.1
Malformations of renal pelvis and ureter	Q62	14	1.1
Other malformations of kidney	Q63	18	1.5
**Musculoskeletal system**	**Q65-Q79**	**230**	**18.8**
Deformities of feet	Q66	38	3.1
Polydactyly	Q69	70	5.7
Other malformations of musculoskeletal system	Q79	69	5.6
**Chromosomal abnormalities**	**Q90-Q99**	**19**	**1.6**
Down's syndrome	Q90	3	0.2
18-trisomy	Q91.0-Q91.3	6	0.5
13-trisomy	Q91.4-Q91.7	7	0.6
**Other malformations**	**Q80-Q89**	**52**	**4.3**
Other known syndrome	Q87	37	3.0
Other malformations	Q89	13	1.1

*Arhinencephaly and microcephaly were not counted as associated abnormalities.

^#^ Arrhinia, single nostril and proboscis were included.

## Discussion

This is a large sample study with data derived from a single register covering both urban and rural population. The prevalence of HPE was 0.92 per 10 000 births in our study. In comparison with some ICBDSR member programs with the same HPE inclusion criteria, our prevalence was comparable to that in Canada (0.94/10,000), higher than that in India (0.59/10,000), and lower than that in Japan (1.06/10,000), USA-Texas (1.19/10,000) and Australia (1.24/10,000) [28]. HPE prevalence in previous studies are difficult to compare with our study directly because of the heterogeneity of inclusion and exclusion criteria, diagnostic capability, and follow-up time. Another source of heterogeneity may be due to the real differences existing among populations. A study in California showed that among cytogenetically normal cases Hispanics had the highest HPE prevalence, followed by Whites, and then Blacks, and Asian population had the lowest HPE prevalence [13]. Our study provides further evidence that lower HPE prevalence exist among Asian populations.

Similar to previous findings of studies in Atlanta [31] and South America [15], an upward trend in HPE prevalence was obtained in our study. In Atlanta, the HPE prevalence increased from 0.44 cases per 10,000 births in 1968–1972 to 1.00 cases per 10,000 births in 1988–1992. Such an increasing trend was also observed in South America (the HPE prevalence after 1996 was nearly twice of the prevalence before 1996). As described in studies by Sun et al. [32, 33], the upward trend of HPE prevalence might be partly ascribed to the improved ascertainment of the abnormality with ultrasound techniques. In addition, the increased proportion of advanced maternal age could also increase the risk of chromosome syndrome as well as the risk of accompanying HPE [7, 34].

Several studies reported that a greater risk of HPE happen among younger women [13, 19]. A U-shaped distribution of HPE prevalence for maternal age was reported in a survey from Atlanta [31]. In the current study, we observed a significant rise of HPE prevalence in advanced maternal age groups (≥ 30 years), but a non-significant rise in younger (<20 years) maternal-age group. Furthermore, urban-rural differences were noted in the prevalence of HPE, that could be due to differences in economic level, maternal environmental exposures, and health care between people who live in urban and rural areas in China [35]. Previous studies identified a female excess in HPE [13, 19, 36], similar result was also found in our study. The reason for the female predominance among HPE cases is unclear. Some studies suggest that female embryos might be more susceptible to HPE, and male embryos with HPE might be more likely to be lost through spontaneous abortion [31, 37].

We have found that 80.7% of HPE cases had additional structural or chromosomal malformations. This is in keeping with data from previous studies. Ong et al. reported that 85% of HPE cases were not isolated, 28% of HPE cases with limb/skeletal defects, 21% and 22% with cardiovascular and urogenital anomalies respectively [16]. Rasmussen et al. reported that 55% of non-syndromic HPE cases were diagnosed with at least one major congenital anomaly [31]. Whiteford et al. found associated anomalies appeared in 36% of HPE cases without chromosome malformation [38]. Chromosomal anomalies, especially trisomy 13 are frequently associated with HPE. In this study, only 1.6% of cases were accompanied by chromosomal disorder, far lower than that reported in other studies [13, 16, 39, 40]. Most Chinese women opt to abort fetuses once anomalies were prenatally diagnosed, but refuse to do further chromosome examination due to economic reasons. There is a great possibility that some chromosomal anomalies were undetected. Thus chromosomal anomalies in association with HPE are likely underestimated in this study.

The survival of HPE infants were strongly correlated with the severity of brain malformation, the presence of chromosomal aberrations or congenital anomalies [2, 11]. Olsen et al. reported a one-year survival rate of 54% for isolated HPE, 25% for HPE with multiple defects, and 14% for syndromic HPE [19]. In the current study, one third of live born infants with isolated HPE and 56.5% of live births affected by multiple anomalies died within the first week of life. Indeed, the overall prognosis of HPE is very poor, which poses a challenge to prenatal and perinatal health care.

### Strengths and limitations

The major strength of this study is the large sample size of subjects which ensure a nationally representative estimate of HPE prevalence owing to the high-quality CBDMN data with wide geographic coverage and consistent ascertainment methods. To our best knowledge, the current study, which included 1222 HPE cases, is the largest epidemiological study on human HPE to date. One limitation is the referral bias introduced by hospital-based samples, which focused on selected hospitals rather than all deliveries in a region. However, the effect could be minimal because the hospital delivery rate reached an average of 99% in China [41]. Newborns delivered in CBDMN member hospitals covered the vast majority of local birth populations. Another limitation is that some structural or chromosomal malformations associated with HPE likely were not detected due to the limited number of chromosomal testing and autopsy. In addition, infants affected by mild HPE might not be identified in the relatively short monitoring period.

## Conclusions

In conclusion, this large study among over ten million births provides robust prevalence estimates for HPE in China. An upward time trend in HPE prevalence was observed during the study period. Explanations for the trend and for the marked subgroup differences in HPE remain unknown. These preliminary findings in our study provide the foundation for future epidemiologic studies in the Chinese population as well as studies in the Asian population in the future.

## Supporting information

S1 File STROBE-checklist. (DOC)

S2 File PLOSOne_Clinical_Studies_Checklist. (DOCX)
